# Associations Between FTO rs9939609 Genotype, Physical Activity, and Dietary Behaviors in Young Adults

**DOI:** 10.3390/nu18101561

**Published:** 2026-05-14

**Authors:** Cassandra Evans, Jaime Tartar, Jonathan Banks, Jennifer Austin McCrae, Jose Antonio

**Affiliations:** 1 Department of Health and Human Performance, Nova Southeastern University, Davie, FL 33328, USA; jose.antonio@nova.edu; 2 Department of Health Science, Rocky Mountain University of Health Professionals, Provo, UT 84606, USA; 3 Department of Psychology and Neuroscience, Nova Southeastern University, Davie, FL 33328, USA

**Keywords:** exercise, FTO, obesity

## Abstract

**Background:** Numerous gene variants are linked to an individual’s propensity to become overweight or obese. A commonly studied gene variant is the FTO single-nucleotide polymorphism (SNP) rs9939609. The FTO risk (A/-) allele associated with this SNP is linked with increased body fat percentage, body mass, BMI, and other lifestyle factors that may perpetuate an individual’s risk for obesity. This study investigated dietary behaviors in individuals engaged in varying levels of physical activity with and without the FTO risk allele. **Methods:** Using a cross-sectional design, adults completed the Three-Factor Eating Questionnaire (TFEQ-R18) and Food Craving Inventory (FCI) to assess dietary behaviors. Body composition was assessed using bioelectrical impedance analysis (in-body analyses). **Results:** Findings indicated that individuals with the FTO risk allele exhibited higher levels of cognitive restraint. No other significant differences were reported in all outcomes between groups. Regression analyses found that physical activity was significantly associated with multiple dietary behaviors (emotional eating, cravings for sweets, the behavioral aspect of fried and sweet cravings), while the FTO risk allele was related to higher cognitive restraint and lower behavioral fried food cravings. **Conclusions:** Combined, these findings indicate that anthropometric measures and most dietary behaviors did not differ across FTO risk variants in physically active individuals, although individuals with higher-risk genotypes exhibited greater cognitive restraint.

## 1. Introduction

The obesity epidemic affects people globally, impacting health and longevity [1,2,3]. An estimated 70% of the US population is overweight or obese based on BMI standards [3]. Increased rates of comorbidities, including cardiovascular disease, type 2 diabetes, and metabolic syndrome, have been observed in individuals suffering from obesity [4]. The manifestation of obesity is complex and involves many factors, such as levels of physical activity, socioeconomic status, environmental conditions, and genetic predispositions.

Genome-wide associated studies (GWASs) have identified more than 300 single-nucleotide polymorphisms (SNPs) linked to obesity. Among genetic variants associated with obesity, SNPs within the FTO locus demonstrate some of the most consistently replicated associations across diverse populations [5,6]. For example, individuals with SNPs occurring on intron-1 of the FTO gene (rs9939609 (A/-), rs17817449 (G/-), rs3751812, rs1421085 (C/-)) have an increased risk of developing obesity [5,6,7,8,9]. Obesity-related traits correlating to the FTO loci can be observed in Western European, Hispanic/Latino, Asian, African American, and Pima Indian populations [6,9,10].

The exact mechanisms by which FTO genetic variants contribute to obesity remain unclear. However, there are demonstrated differences in anthropometric measures between individuals with and without the risk allele in FTO SNP [11]. Higher BMI, body mass, body fat percentage, and abdominal adipose tissue are associated with polymorphisms of the FTO gene [5,12,13,14]. Interestingly, similar differences in anthropometric measurements are found in exercise-trained individuals [13].

Dietary behaviors is a broad term that refers to cognitive and behavioral processes that shape eating patterns [15]. These behaviors can be either adaptive or maladaptive depending on context, frequency, and degree. Commonly studied behaviors include uncontrolled eating (UE), emotional eating (EE), cognitive restraint (CR), and food cravings [16]. UE involves overeating with a perceived loss of control, while EE reflects eating in response to negative emotional states. Both EE and UE are generally considered maldapative [17]. CR refers to the deliberate restriction of food intake, regardless of physiological hunger cues [16]. Food cravings are “an intense desire to consume a particular food or food type that is difficult to resist” and may occur without hunger or the need for food [18,19]. Food cravings occur spontaneously (tonic) or as the result of an environmental/external stimulus (cue-induced) [19]. Dietary behaviors influence caloric intake and anthropometrics [16]. Imbalances, deficiencies, or excess intake result in changes in weight and health. Higher intakes of sugar, saturated fats, and ultra-processed foods are linked to an increased risk of cardiovascular disease [20,21].

The FTO rs9939609 SNP is involved with the regulation of energy intake. Individuals with the risk (A/-) allele have higher energy intakes, specifically from fats and sugars, and they experience lower levels of satiety [22,23,24,25,26,27,28,29,30,31,32,33,34]. As mentioned previously, simply carrying a risk allele does not dictate obesity. However, the combination of increased energy intake, lower levels of satiety, and an obesogenic environment contribute to weight gain and the health status of those with a higher risk. Differences in eating behaviors, food preferences, and satiety are also reported in individuals with varying levels of physical activity [35,36,37]. Individuals who regularly engage in physical activity exhibit higher levels of healthy eating behaviors [38,39,40,41,42]. Healthy eating behaviors include greater consumption of nutrient-dense foods such as fruits, vegetables, and whole foods, along with a lower intake of energy-dense snacks, fast foods, and sugar-sweetened products [43]. Levels of emotional eating, body dissatisfaction, and consumption of high fat, snack, and fast foods are lower in those engaged in physical activity, even during times of increased stress [40,41]. Conversely, sedentary behavior is linked to higher levels of emotional eating, uncontrolled eating, and overconsumption [35,36,37].

Most studies investigating FTO polymorphisms have been conducted in overweight or obese populations, with fewer studies examining these relationships in physically active individuals [5,6,22,23,24,25,26,27,28,29,30,31,32,33,34]. As a result, it remains unclear whether differences in dietary behaviors and anthropometric outcomes persist in individuals who engage in regular physical activity or whether physical activity is related to the phenotypic expression of FTO genetic variants. In order to address this gap in the literature, the current study examined dietary behaviors across varying levels of physical activity in FTO rs9939609 risk allele carriers (A/-) versus non-carriers (TT). We hypothesized that differences in anthropometrics and eating behaviors between risk groups will still be present in healthy individuals engaged in regular physical activity.

## 2. Materials and Methods

The current study used a cross-sectional design to examine the interactions between the level of physical activity, dietary behaviors, and the FTO risk allele. Subjects were recruited from the Nova Southeastern University campus located in Davie, Florida. Individuals were excluded if they had a history of eating disorders or were actively engaged in weight loss efforts. All participants provided informed consent in accordance with the Declaration of Helsinki and an approved IRB protocol submitted to the institutional review boards of Nova Southeastern University and the Rocky Mountain University of Health Professionals (IRB# 2024-446). Subjects were instructed to arrive at the laboratory following their usual daily routine. During the testing session, subjects underwent testing for body composition, provided a saliva sample, and completed an online survey consisting of demographic questions, TFEQ-18, and the FCI.

### 2.1. Anthropometrics

Anthropometrics were assessed using methods previously described by Evans et al. [44]. Body composition was assessed using a multi-frequency bioelectrical impedance device (InBody^®^, Cerritos, CA, USA). The InBody bioelectrical impedance analyzer (BIA) is a validated and reliable tool commonly used in research settings to assess body composition, including fat mass, lean body mass, and total body water [45]. Participants were instructed to abstain from food, liquids, and strenuous activity for at least 4 h prior to testing to reduce measurement variability. Study participants stood on the device’s platform barefoot with the soles of their feet on the electrodes and then grasped the unit’s handles with their thumb and fingers to maintain direct contact with the electrodes. They stood still for ~1 min with their elbows fully extended and their shoulder joints at a ~30-degree angle.

### 2.2. Physical Activity

Physical activity was assessed via self-reporting. Participants reported the number of minutes per week they engaged in cardiovascular exercise and strength training. Total physical activity (PA Total) was calculated as the sum of weekly minutes of cardiovascular and strength exercise. No standardized or validated physical activity questionnaire was used.

### 2.3. Dietary Behaviors

Dietary behaviors were assessed using two questionnaires: the Three-Factor Eating Questionnaire (TFEQ-18) and the Food Craving Inventory (FCI).

The TFEQ-R18 consists of 18 questions assessing cognitive restraint, emotional eating, and uncontrolled eating. The instrument is a shortened and revised version of the original 51-item TFEQ and considered both valid and reliable [46]. Participants responded to a series of 18 items on a scale from 1 (definitely true) to 4 (definitely false), and item scores were summed into scale scores for cognitive restraint, uncontrolled eating, and emotional eating. Raw scale scores for each subscale were converted to a 0–100 scale, as previously described by de Lauzon et al. [46].

The FCI is a previously validated self-reported survey assessing general and specific food cravings. This assessment differs from other assessments as it gathers information about specific foods rather than an individual’s subjective experience. Participants are asked about cravings for specific foods over the last month. A follow-up question asks how often they consumed the craved food as a measure of consumption [47]. Answers are recorded using a Likert scale from 1 (never) to 5 (always/almost every time). The measure has four subscales: fried foods, dietary fats, sweets, and starches [18]. The average of each subscale is calculated and higher score on a subscale or total score indicates a greater frequency of craving for that category of food.

### 2.4. Genotyping

Genotyping was assessed using methods previously described by Antonio et al. [13]. Genomic DNA was extracted using a QIAcube instrument following the manufacturer’s standard protocol for saliva nucleic acid extraction (QIAGEN, Valencia, CA, USA). After isolation, allelic discrimination for the FTO SNPs was determined via real-time polymerase chain reaction (PCR) using TaqMan SNP genotyping assays using fluorogenic probes (Applied Biosystems, Foster, CA, USA) with the following primer sequences:

rs9939609:

GGTTCCTTGCGACTGCTGTGAATTT [A/T] GTGATGCACTTGGATAGTCTCTGTT.

Thermal cycling was performed on the StepOne Real-Time PCR system (Applied Biosystems, CA). The amplification mix contained the following ingredients: 12.5 μL of PCR master mix (QIAGEN, Valencia, CA, USA), 1.25 μL of TaqMan 20× working stock, 10.25 μL of RNase- and DNase-free water (Sigma-Aldrich, St. Louis, MO, USA), and 1.0 μL of sample DNA in a total volume of 25 μL per single tube reaction. The PCR condition was 95 °C for 10 min followed by 40 repeated cycles of 95 °C for 15 s and 60 °C for 60 s. Genotypes were determined automatically via StepOne software V2.3 (Applied Biosystems, CA) based on the fluorescence signals. Samples were run in duplicate and in the case of a call discrepancy, samples were rerun. Participants with normal obesity risk were classified as “non-risk allele” versus those that carried the risk as “risk allele”. We classified those with normal obesity risk (T/T) as “non-risk allele” versus those that carried the risk as “risk allele” (A/T and A/A) (i.e., both heterozygous and homozygous).

### 2.5. Statistical Analysis

All statistical analyses were conducted using R version 4.5.0 (11 April 2025). Descriptive statistics were computed for all variables. A two-tailed independent samples *t*-test was conducted to examine risk differences in dietary outcomes and body composition between groups. Welch’s *t*-test was employed for all comparisons by risk, as it does not assume equal variances or sample sizes [48,49]. The significance level was at *p* < 0.05, and all *t*-tests used a two-tailed *p*-value. A Pearson correlation was conducted to assess any relationships between body fat percentage, risk, and dietary behavior outcomes prior to multivariable analysis. Multiple linear regression analyses were performed to examine associations between FTO risk status and dietary behavior outcomes. Separate models were conducted for each outcome, including uncontrolled eating, cognitive restraint, emotional eating, total food craving score, and subscales for fat, sweets, carbohydrates, and fried food cravings, as well as their corresponding behavioral measures. All models included total physical activity, sex, age, and body fat percentage as covariates. Assumptions for linear regression were evaluated using standard diagnostic plots. A visual inspection of residual, Q–Q, and scale-location plots indicated no major deviations from linearity, normality, or homoscedasticity. Multicollinearity was assessed using variance inflation factors (VIFs), with all values indicating no concerns.

## 3. Results

All participants were genotyped for FTO SNP rs9939609; 106 (61.6%) were categorized as possessing the obesity risk allele (AA or AT) and 66 (38.4%) were categorized as having normal or no risk (TT). Descriptive statistics for age, height, body mass, BMI, body fat percentage, fat mass, LBM, and total physical activity for the week can be found in Table 1. All variables met the assumptions of normal distribution.

### 3.1. Risk Differences

To determine whether there were differences between individuals with and without the risk allele, we conducted a two-tailed independent samples *t*-test. A significant difference between those with the risk and those without was observed for one of the TFEQ-18 subscales. Individuals with the risk allele exhibited higher levels of cognitive restraint (*p* = 0.011, *d* = −0.4). No other significant differences were reported in anthropometric and dietary behaviors, as seen in Table 2.

### 3.2. Adjusted Differences in Dietary Behaviors

To examine associations between FTO risk status and dietary behaviors after adjusting for relevant covariates, multiple linear regression analyses were conducted including total physical activity, sex, age, and body fat percentage. Physical activity was significantly associated with several dietary behaviors, including cognitive restraint (β = 0.0136, F(5,165) = 6.50, *p* = 0.009), sweet cravings (β = −0.00042, F(5,165) = 2.81, *p* = 0.016), fried food cravings (β = −0.00034, F(5,165) = 1.69, *p* = 0.046), and their corresponding behavioral components.

Body fat percentage was significantly associated with cognitive restraint (β = 0.881, F(5,165) = 6.50, *p* < 0.001), emotional eating (β = 0.787, F(5,165) = 6.27, *p* < 0.001), and sweet cravings (β = −0.0164, F(5,165) = 2.81, *p* = 0.021).

FTO risk status was significantly associated with higher cognitive restraint (β = 8.95, F(5,165) = 6.50, *p* = 0.011) and lower behavioral fried food cravings (β = −0.249, F(5,164) = 3.05, *p* = 0.048), with no significant associations observed for other dietary outcomes.

### 3.3. Pearson Correlation by Risk

In the risk group, body fat percentage was positively associated with cognitive restraint and emotional eating and inversely associated with total physical activity. Across both groups, uncontrolled eating was strongly correlated with emotional eating, total food cravings, and specific cravings for sweets, carbohydrates, and fried foods. In the risk group, emotional eating was positively correlated with cognitive restraint, fried food cravings, and the behavioral component of sweet intake, whereas these associations were not significant in the no-risk group. Across both groups, total food cravings were strongly correlated with fat, sweet, carbohydrate, and fried food cravings, as well as their corresponding behavioral components (Table 3).

## 4. Discussion

The present study did not observe any differences in anthropometrics in physically active individuals with and without the FTO risk. Importantly, these findings remained unchanged after controlling for physical activity, sex, age, and body fat percentage. This indicates that the lack of group differences was not explained by variation in activity levels, sex, age or body fat percentage. The following interpretations should be considered as potential explanations of the observed associations, as the present study did not directly assess underlying behavioral or physiological mechanisms. Those with the FTO risk exhibited higher cognitive restraint than those without. Although no differences in the levels of physical activity between risk groups were observed, a Pearson correlation analysis revealed a relationship between physical activity and body composition only in those with the FTO risk.

The FTO risk allele is correlated with higher levels of anthropometric variables (body fat, BMI, body mass) across various populations [12,13,14,50]. Conversely, the present study did not find any significant differences in anthropometrics between risk groups. These findings differ from Antonio et al.’s study [13] (2018), where exercise-trained individuals with the risk allele had significantly greater fat mass and body fat percentage. Participants in the Antonio et al. [13] (2018) study were notably leaner than the present study population. It is possible that the effects of FTO risk may be more pronounced in highly lean individuals, where small variations in body composition are more detectable. In the present study, participants reported relatively high levels of physical activity, with average weekly cardiovascular exercise exceeding commonly recommended minimum thresholds [51]. Thus, the high level of exercise may account for the lack of differences observed in anthropometrics. However, the intensity and frequency (e.g., number of days per week) of physical activity were not assessed, limiting a direct confirmation of adherence to the current ACSM guidelines. Prior research suggests physical activity lowers the risk for obesity despite possessing the FTO risk allele [28,52,53,54,55]. Kim et al. [52] reported that subjects who were regularly engaged in active physical activity had a lower BMI and weight irrespective of possessing the risk allele. West et al. [54] reported high levels of physical activity and no differences in anthropometrics between risk groups. The study used self-reported data in all outcomes, while the present study used bioelectrical impedance, which enables the authors to include accurate values for BMI, body fat percentage, and fat mass. Similar to this study, self-reported physical activity was collected, which is a limitation due to the tendency for subjects to overestimate activity. While the findings of the present study are consistent with this broader pattern observed in the current literature [28,52,53,54,55], the cross-sectional design does not allow for a direct assessment of interaction or causal effects.

Despite this limitation, our findings are supported by a meta-analysis of 528 studies which found a beneficial effect of physical activity in individuals with FTO risk. Kilpeläinent et al. [53] estimates a 30% decrease in obesity risk in physically active individuals compared to sedentary individuals. Interestingly, the meta-analysis found a stronger interaction in North America compared to other locations. The authors theorize higher levels of sedentary behaviors, which are associated with unhealthy dietary behaviors and are more common in North America, account for this difference.

In the present study, no group differences in anthropometric outcomes were observed; however, a significant inverse association between physical activity and body fat percentage was identified in the FTO risk group only. Similarly, Andreasen et al. [12] reported a correlation between physical inactivity and increased body mass in individual with the risk allele but not in the non-risk group. Based on these findings, the authors theorize that the relationship between physical activity and body composition may differ by FTO genotype.

Individuals with the risk allele are more susceptible to hunger, emotional eating, and a higher intake of unhealthy foods, which may contribute to obesity risk [24,34,56]. The present study observed limited differences in dietary outcomes. Dang et al. [56] reported higher total food craving scores in the individuals with FTO risk. Typically, food cravings decrease with age; however, this was only observed in individuals without FTO risk. Harbon et al. [24] reported that individuals with the risk allele (variants rs17817449 and rs1421085) exhibited higher intakes of high-fat foods and refined starches, and they experienced increased hunger and emotional disinhibition. It is important to note that dietary behaviors are influenced by a variety of factors and observed associations may reflect situational influences rather than stable behavioral patterns.

In the present study, the risk group exhibited significantly higher cognitive restraint scores even after controlling for physical activity. Cognitive restraint refers to the intentional restriction of food intake to control body weight or appearance and can be characterized as either flexible or rigid [57]. Flexible restraint is generally associated with more favorable BMI outcomes, whereas rigid restraint has been linked to maladaptive eating behaviors and increases in body fat and/or body mass [58]. Consistent with these distinctions, Harbon et al. [24] reported higher levels of both self-regulation and rigid restraint in individuals with the risk allele. However, an inverse relationship between cognitive restraint and BMI was observed only in non-risk individuals, which may reflect the more adaptive nature of flexible restraint, as a higher BMI is more commonly associated with rigid restraint. Similarly, higher levels of cognitive restraint in individuals with the risk allele have been reported in physically active populations [54]. In the present study, the risk group also exhibited significantly higher cognitive restraint after controlling for physical activity; however, the type of cognitive restraint (i.e., flexible vs. rigid) was not assessed in this study. Collectively, these findings suggest the possibility that individuals with the FTO risk allele may exert greater effort to regulate their food intake, potentially as a compensatory response to heightened hunger and food cravings, although this mechanism was not directly assessed in the present study.

Although the literature largely demonstrates genotype-related differences in dietary behaviors, some studies have reported no differences [26,50]. Physical activity may partially explain these findings. Melhourn et al. [26] assessed physical activity using the IPAQ and categorized participants as having moderate-to-high activity levels across both groups. While the risk group exhibited lower satiety and higher caloric intake in a buffet setting, no differences in eating behaviors were observed using the TFEQ−18. Although physical activity was not a primary focus, the relatively high activity levels of participants may be associated with differences in eating behaviors typically associated with the FTO risk allele. Similarly, West et al. [54] reported similar scores between risk groups in physically active individuals on the TFEQ-18 subscales for disinhibition and emotional eating.

In the present study, higher levels of physical activity were related to lower levels of emotional eating, fewer cravings for sweets, and less consumption of fried foods in response to cravings independent of risk. These results align with prior research linking regular exercise to healthier dietary behaviors. Studies conducted during the COVID-19 pandemic similarly reported a protective effect of physical activity [40,41]. Compared to sedentary individuals, those who regularly engaged in physical activity exhibited lower levels of emotional eating and body dissatisfaction, as well as a reduced consumption of high-fat foods, snacks, and fast foods, despite increased external stressors associated with the pandemic.

Regular physical activity is associated with lower obesity risk in those with the FTO risk allele [28,52,53,54,55]. Additionally, regular physical activity is associated with more favorable dietary behaviors, such as improved appetite regulation, reduced emotional eating, and healthier food choices, irrespective of obesity risk [59]. These behavioral adaptations likely contribute to the maintenance of healthy body weight and anthropometric outcomes. Given the increased susceptibility to maladaptive eating patterns in individuals with the risk allele, physical activity may influence these behaviors.

In the present study, a Pearson correlation analysis revealed an inverse relationship between physical activity and body composition, as well as the consumption of sweets in response to a craving. Interestingly, this correlation was not observed in participants without risk. This finding suggests that the impact of physical activity on dietary behaviors and body composition may possibly be more pronounced in individuals with a genetic susceptibility to obesity. Similarly, emerging evidence suggests that other lifestyle behaviors, including sleep and eating patterns, may interact with the FTO genotype. Kazarnovsky Nahshan et al. [60] found that favorable lifestyle behaviors, including sleep and eating patterns, decreased the risk of type 2 diabetes associated with the FTO rs9939609 risk allele. The authors postulated that the beneficial effect of exercise may be more pronounced in individuals with an increased risk for obesity. A study conducted by Danahar et al. [61] found lower FTO mRNA expression following bouts of high-intensity exercise, suggesting that the effects of exercise could occur at both the molecular level and through potential improvements in dietary behaviors.

This study has several limitations. First, several outcomes relied on self-reported data, which are subject to recall bias, reporting inaccuracies, and social desirability bias. These limitations may be particularly relevant for dietary behaviors and physical activity, where over- or under-reporting could influence observed associations. Additionally, some items on the FCI are animal-based, which may limit its accuracy in assessing food cravings among those following a plant-based diet. Although no upper age limit was set for the current study, most of the participants were young, physically active university students, limiting generalizability to broader populations. Lifestyle behaviors typically differ in college due to factors including financial constraints, accessibility, and being away from home [62]. The predominance of null findings across most outcomes may reflect the relatively homogeneous and physically active nature of the sample compared to more diverse or higher-risk populations (e.g., sedentary or overweight individuals), where FTO-related effects are more consistently reported. Multiple dietary and behavioral outcomes were examined, which increases the risk of type I error; therefore, isolated significant findings should be interpreted with caution, although the observed association for cognitive restraint is consistent with prior research.

## 5. Conclusions

Physical activity supports overall health through its effects on anthropometrics and dietary behaviors. Sedentary lifestyles are associated with poorer dietary habits and increased obesity risk. Similarly, FTO risk is associated with higher body mass and unhealthy dietary habits, which increase an individual’s risk for obesity. In the present study, the FTO genotype was not associated with differences in dietary behaviors and anthropometrics typically associated with possessing the FTO risk allele. These findings suggest that, within physically active young adult populations, behavioral factors such as physical activity may play a role in shaping dietary patterns independent of the FTO genotype. Although genetics predispose individuals to certain behaviors, lifestyle modifications, specifically engaging in physical exercise, appears to be associated with differences in obesity-related outcomes across FTO genotypes. Given the cross-sectional design, reliance on self-reported measures, and limited sample diversity, these findings should be interpreted with caution.

## Figures and Tables

**Table 1:** Descriptive statistics.

	Total (*n* = 172)			No Risk (*n* = 66)			Risk (*n* = 106)		
	Mean		Std. Deviation	Mean	Std. Deviation		Mean	Std. Deviation	
Age	21.08	±	4.498	20.58	±	2.643	21.39	±	5.327
Sex	0.65	±	0.478	0.59	±	0.495	0.69	±	0.465
Height (cm)	169.579	±	10.0389	169.927	±	8.3595	169.362	±	10.9883
Body Mass (kg)	70.798	±	16.404	72.226	±	14.5699	69.908	±	17.4568
BMI	24.741	±	4.3015	24.948	±	4.2747	24.611	±	4.3333
Body Fat (%)	24.767	±	9.9581	23.821	±	9.9722	25.356	±	9.9509
Fat Mass (kg)	17.945	±	8.9333	17.561	±	9.4588	18.185	±	8.6273
LBM (kg)	53.455	±	13.1879	54.658	±	11.7397	52.707	±	14.016
Strength (min)	201.21	±	194.281	222.14	±	204.471	188.41	±	187.641
Cardio (min)	178.21	±	223.434	192.08	±	262.69	169.41	±	195.354
PA_Total (min)	370.33	±	333.848	404.12	±	373.398	349.29	±	306.652

LBM—lean body mass; PA_Total—total physical activity.

**Table 2:** Risk differences.

	No Risk (*n* = 66)			Risk (*n* = 106)				95% Confidence Interval of the Difference			
	Mean		Std. Dev	Mean		Std. Dev	t-Value	Lower	Upper	*p*-Value	Cohen’s d
Body Mass	72.226	±	14.570	69.908	±	17.457	0.939	−2.558	7.192	0.349	0.14
BMI	24.948	±	4.275	24.611	±	4.333	0.500	−0.995	1.669	0.618	0.08
BodyFat	23.821	±	9.972	25.356	±	9.951	−0.982	−4.624	1.555	0.328	−0.15
Fat Mass	17.561	±	9.459	18.185	±	8.627	−0.435	−3.463	2.214	0.664	−0.07
LBM	54.658	±	11.740	52.707	±	14.016	0.983	−1.971	5.873	0.327	0.15
UE	47.236	±	18.227	50.105	±	16.849	−1.028	−8.391	2.654	0.306	−0.17
CR	43.504	±	22.191	52.715	±	23.793	−2.563	−16.315	−2.106	0.011	−0.4
EE	23.761	±	24.991	28.092	±	22.756	−1.138	−11.866	3.203	0.257	−0.18
FCI	9.456	±	2.178	9.257	±	2.029	0.596	−0.462	0.861	0.552	0.1
Fat	2.076	±	0.600	1.919	±	0.533	1.728	−0.023	0.336	0.087	0.28
Sweet	2.298	±	0.701	2.473	±	0.775	−1.519	−0.402	0.053	0.131	−0.23
CHO	2.382	±	0.690	2.236	±	0.645	1.382	−0.063	0.356	0.170	0.22
FriedFood	2.700	±	0.805	2.629	±	0.657	0.599	−0.164	0.306	0.550	0.1
FCI_B	8.422	±	2.823	7.995	±	2.213	1.046	−0.382	1.237	0.298	0.17
Fat_B	1.842	±	0.629	1.692	±	0.563	1.570	−0.039	0.340	0.119	0.26
Sweet_B	2.038	±	0.744	2.037	±	0.717	0.012	−0.227	0.230	0.990	0
CHO_B	2.172	±	0.698	1.965	±	0.656	1.918	−0.007	0.421	0.057	0.31
Fried_B	2.562	±	0.895	2.302	±	0.740	1.964	−0.002	0.522	0.052	0.32

LBM—lean body mass; UE—uncontrolled eating; CR—cognitive restraint; EE—emotional eating; FCI—Food Craving Inventory score; CHO—carbohydrate; _B denotes behavioral aspects of respective craving.

**Table 3:** Correlations (data for risk are presented below the diagonal and data for no risk are presented above the diagonal).

	Sex	BodyFat	LBM	Strength	Cardio	PA_Total	UE	CR	EE	FCI	Fat	Sweet	CHO	FriedFood	FCI_B	Fat_B	Sweet_B	CHO_B	Fried_B
Sex		**0.614**	**−0.848**	−0.115	0.198	0.075	−0.028	0.084	0.149	−0.204	**−0.36**	0.017	−0.117	−0.198	−0.141	**−0.402**	−0.074	−0.212	−0.217
BodyFat	**0.528**		**−0.47**	−0.122	−0.017	−0.067	0.008	0.232	0.177	−0.176	−0.234	−0.061	−0.17	−0.102	−0.037	−0.209	−0.002	−0.211	−0.036
LBM	**−0.755**	**−0.484**		0.138	−0.135	−0.019	0.082	−0.075	−0.103	0.205	**0.305**	−0.007	0.18	0.178	0.116	**0.321**	0.092	0.212	0.202
Strength	**−0.284**	**−0.35**	**0.327**		**0.25**	**0.723**	0.072	0.064	−0.137	0.013	0.089	−0.049	0.24	−0.194	0.011	0.101	−0.104	0.193	−0.105
Cardio	0.087	−0.162	−0.018	**0.27**		**0.848**	−0.005	0.098	−0.091	−0.167	−0.182	−0.161	0.057	−0.225	−0.078	−0.105	−0.116	0.048	−0.213
PA_Total	−0.109	**−0.289**	0.185	**0.787**	**0.807**		0.02	0.102	−0.15	−0.133	−0.089	−0.171	0.139	**−0.262**	−0.049	−0.009	−0.141	0.137	−0.203
UE	−0.12	0.021	0.124	0.103	0.045	0.086		0.061	**0.63**	**0.415**	0.196	**0.397**	**0.423**	**0.268**	**0.321**	0.214	**0.35**	**0.28**	0.224
CR	0.182	**0.352**	−0.025	0.156	0.057	0.139	0.121		0.03	−0.187	−0.166	−0.104	−0.204	−0.117	−0.216	−0.219	**−0.259**	−0.216	−0.082
EE	**0.284**	**0.512**	**−0.311**	−0.189	−0.089	−0.169	**0.467**	**0.218**		0.21	0.056	**0.43**	0.165	0.01	0.158	0.033	**0.356**	0.101	0.035
FCI	−0.161	−0.146	0.04	0.111	−0.174	−0.075	**0.33**	−0.108	0.146		**0.784**	**0.741**	**0.796**	**0.793**	**0.823**	**0.613**	**0.751**	**0.682**	**0.723**
Fat	**−0.368**	**−0.267**	**0.253**	**0.226**	−0.108	0.05	**0.357**	−0.189	0.058	**0.791**		**0.418**	**0.596**	**0.502**	**0.731**	**0.825**	**0.555**	**0.605**	**0.554**
Sweet	0.006	−0.112	−0.03	−0.026	−0.182	−0.151	**0.272**	−0.038	0.13	**0.803**	**0.505**		**0.451**	**0.436**	**0.492**	0.223	**0.725**	**0.257**	**0.386**
CHO	−0.114	−0.128	0.027	0.147	−0.117	−0.034	**0.211**	0.003	0.029	**0.768**	**0.516**	**0.491**		**0.458**	**0.68**	**0.499**	**0.519**	**0.822**	**0.483**
FriedFood	−0.093	0.025	−0.073	0.051	−0.117	−0.062	**0.203**	−0.137	**0.221**	**0.744**	**0.528**	**0.409**	**0.393**		**0.67**	**0.389**	**0.544**	**0.431**	**0.793**
FCI_B	−0.122	−0.134	0.016	−0.082	−0.146	−0.158	0.129	**−0.368**	0.087	**0.624**	**0.579**	**0.408**	**0.474**	**0.509**		**0.86**	**0.844**	**0.851**	**0.88**
Fat_B	**−0.193**	−0.188	0.105	−0.037	−0.137	−0.119	0.115	**−0.353**	0.041	**0.49**	**0.66**	**0.253**	**0.318**	**0.365**	**0.866**		**0.639**	**0.734**	**0.638**
Sweet_B	0.031	−0.081	−0.063	−0.173	−0.149	**−0.21**	0.168	**−0.286**	0.128	**0.575**	**0.442**	**0.63**	**0.321**	**0.359**	**0.832**	**0.645**		**0.589**	**0.674**
CHO_B	−0.122	−0.163	0.044	0.042	−0.072	−0.057	0.058	**−0.219**	−0.038	**0.548**	**0.444**	**0.288**	**0.729**	**0.276**	**0.767**	**0.595**	**0.48**		**0.634**
Fried_B	−0.139	−0.036	−0.009	−0.084	−0.125	−0.128	0.084	**−0.361**	0.139	**0.45**	**0.408**	0.161	**0.218**	**0.653**	**0.847**	**0.677**	**0.604**	**0.49**	

Note: All correlations presented in bold are significant at the *p* < 0.05 level. UE—uncontrolled eating; CR—cognitive restraint; EE—emotional eating; FCI—Food Craving Inventory score; CHO—carbohydrate; _B denotes behavioral aspects of respective craving.

## Data Availability

The original contributions presented in this study are included in the article. Further inquiries can be directed to the corresponding author.
